# Trends in breast, colon, pancreatic, and uterine cancers in women during the COVID‐19 pandemic in North Carolina

**DOI:** 10.1002/cam4.7156

**Published:** 2024-04-04

**Authors:** Sarah J. Nyante, Allison M. Deal, Hillary M. Heiling, Kyung Su Kim, Cherie M. Kuzmiak, Benjamin C. Calhoun, Emily M. Ray

**Affiliations:** ^1^ Department of Radiology University of North Carolina at Chapel Hill Chapel Hill North Carolina USA; ^2^ Lineberger Comprehensive Cancer Center University of North Carolina at Chapel Hill Chapel Hill North Carolina USA; ^3^ Department of Pathology and Laboratory Medicine University of North Carolina at Chapel Hill Chapel Hill North Carolina USA; ^4^ Division of Oncology, Department of Medicine University of North Carolina at Chapel Hill Chapel Hill North Carolina USA

**Keywords:** breast cancer, colon cancer, epidemiology, pancreatic cancer, prognosis, uterine cancer, women

## Abstract

**Importance:**

The COVID‐19 pandemic led to reductions in primary care and cancer screening visits, which may delay detection of some cancers. The impact on incidence has not been fully quantified. We examined change in cancer incidence to determine how the COVID‐19 pandemic may have altered the characteristics of cancers diagnosed among women.

**Methods:**

This study included female patients aged ≥18 years and diagnosed with breast (*n* = 9489), colon (*n* = 958), pancreatic (*n* = 669), or uterine (*n* = 1991) cancer at three hospitals in North Carolina. Using interrupted time series, we compared incidence of cancers diagnosed between March 2020 and November 2020 (during pandemic) with cancers diagnosed between January 2016 and February 2020 (pre‐pandemic).

**Results:**

During the pandemic, incidence of breast and uterine cancers was significantly lower than expected compared to pre‐pandemic (breast—18%, *p* = 0.03; uterine −20%, *p* = 0.05). Proportions of advanced pathologic stage and hormone receptor‐negative breast cancers, and advanced clinical stage and large size uterine cancers were more prevalent during the pandemic. No significant changes in incidence were detected for pancreatic (−20%, *p* = 0.08) or colon (+14%, *p* = 0.30) cancers.

**Conclusion and Relevance:**

In women, the COVID‐19 pandemic resulted in a significant reduction in the incidence of breast and uterine cancers, but not colon or pancreatic cancers. A change in the proportion of poor prognosis breast and uterine cancers suggests that some cancers that otherwise would have been diagnosed at an earlier stage will be detected in later years. Continued analysis of long‐term trends is needed to understand the full impact of the pandemic on cancer incidence and outcomes.

## Graphical abstract

Breast and uterine cancer incidence was significantly lower than expected during the first months of the pandemic, suggesting that a significant number of breast and uterine cancers went undiagnosed during that time. Delayed diagnosis of cancers with better prognostic features may result in a shift to less treatable cancers diagnosed in subsequent years.

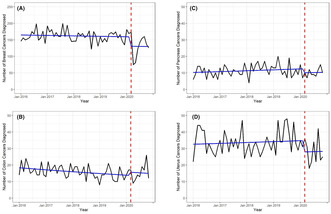

## 1 INTRODUCTION

The COVID‐19 pandemic disrupted cancer care, with US institutions reporting 80%–99% reductions in mammography and 45%–86% reductions in colon cancer screenings at the height of pandemic restrictions.^1^, ^2^, ^3^, ^4^, ^5^, ^6^, ^7^, ^8^, ^9^, ^10^, ^11^ Prior to the pandemic, a majority of eligible women participated in recommended cancer screening. As of 2018, 66% of women >40 years old had a recent mammogram and 64% of women >50 years old had guideline concordant colon cancer screening.^12^, ^13^ Thus, pandemic‐related screening reductions have the potential to impact millions of women and translate into a difference in early cancer detection. Data from our group and others indicate that not all women completed examinations that were initially delayed or canceled within the first 6 months of the pandemic.^7^, ^8^ It is likely that the population of women who missed exams is composed of those who deferred care by a few weeks or months and others who delayed longer or elected to skip their exam altogether. As a result, the pandemic delayed cancer detection for an unknown proportion of women and for an unknown period of time.

Early data has shown that the number of new breast and colon cancer diagnoses fell substantially during the first months of the pandemic,^9^, ^14^, ^15^, ^16^ but data related to the characteristics of the cancers diagnosed during the pandemic are still limited. In this study, we report trends in the incidence of breast cancers among women in a North Carolina Health system between 2016 and 2020. As a comparison, we also report on trends in colon, pancreatic, and uterine cancers during the same time period to explore the degree to which the effects of the pandemic may be influenced by the availability of screening, patterns of gynecologic preventive care, or other factors. Information about how the incidence of these cancers has changed during the pandemic is essential for understanding what the population of Covid‐era cancer survivors will look like as well as inform predictions of the number of excess cancer deaths that may be associated with the pandemic.

## 2 METHODS

### 2.1 Study population

In this study, we evaluated incidence of breast, colon, pancreatic, and uterine cancers diagnosed among women aged ≥18 years at one academic medical center and two community‐based hospitals within the University of North Carolina (UNC) Health Network between January 2016 and November 2020 (the latest date for which tumor registry data were available at the time of the analysis). Cancers were identified using topographic codes (breast: C500‐C506, C508‐C509; colon: C180, C182‐C189; pancreas: C250‐C254, C257‐C259; and uterus: C540‐C543, C548‐C549, C559). Breast cancers included invasive tumors and ductal carcinoma in situ. Uterine cancers included uterine corpus cancers only; cancers of the cervix were excluded due to low numbers. Diagnoses that occurred between January 1, 2016 and February 29, 2020 were classified as “pre‐pandemic,” and diagnoses that occurred between March 1, 2020 and November 30, 2020 were classified as “during pandemic.”

### 2.2 Disease characteristics

All data were abstracted by certified tumor registrars as part of routine cancer documentation at each hospital, and included tumor size, number of lymph nodes evaluated, number of positive lymph nodes, American Joint Committee on Cancer (AJCC) stage at diagnosis, and for breast cancer, receptor status. Stage at diagnosis included AJCC 7th and 8th edition assessments according to diagnosis year. For breast cancer, estrogen receptor (ER) and progesterone receptor (PR) were classified as positive if quantitative expression was recorded as ≥1% or Allred score ≥3. If quantitative expression was not recorded, expression was classified as positive if the summary variable was coded as positive or borderline. Human epidermal growth factor receptor 2 (HER2) was classified as positive if expression was positive by immunohistochemistry or in situ hybridization assays and classified as negative if immunohistochemistry or in situ hybridization results were equivocal or negative. Breast cancer pathologic prognostic stage at diagnosis was classified based on AJCC pathologic stage, ER, PR, HER2, and tumor grade.^17^

### 2.3 Statistical analysis

We tabulated monthly counts for each cancer and tested for seasonal autocorrelation by calculating the Durbin–Watson statistic, where we modeled the counts with time (in months) for the pre‐pandemic time period (January 2016 to February 2020).^18^, ^19^ The 12‐month autocorrelation tests were not statistically significant at *α* = 0.05, and we concluded that no additional seasonality adjustments were required. For each cancer site, we used interrupted time series (ITS) analyses to compare the trends in monthly incidence in pre‐pandemic versus pandemic time periods. The ITS analyses were evaluated using traditional regression techniques and included two covariates: a variable for the month (values 1–59, representing January 2016 to November 2020) and an indicator of whether the month was in the “during pandemic” period, defined from March 2020 to November 2020. The average rate of decrease in the number of subjects diagnosed each month during pandemic compared to the expected number of based on the pre‐pandemic trend was modeled using log‐linear (Poisson) regression. Associated 95% confidence intervals (CIs) and *p*‐values were estimated using a robust variance estimator. The average change in the monthly proportion of cancers with specific poor‐prognosis characteristics (e.g., proportion of ER‐negative breast cancers each month) during the pandemic compared to pre‐pandemic was estimated using linear regression. Poor prognosis tumor size was defined based on the 80th percentile among cases diagnosed in the pre‐pandemic period for breast and uterine cancers. Advanced breast cancer was defined as pathologic prognostic stage II or higher.^17^

The first months of the pandemic included multiple changes that may have affected the ability to access healthcare (Table S1). As a sensitivity analysis, we estimated the change in incidence trends when defining the pandemic period as March 2020 to August 2020 (i.e., truncating the data at August 31, 2020), based on the fact that cancer screening utilization was reduced during this time,^8^, ^11^ or as April 2020 to November 2020 to account for the fact that pandemic disruptions to cancer diagnoses may have lagged behind the disruptions to the screening and diagnostic visits that would lead to a diagnosis. We also repeated analyses restricting the study population to individuals who were diagnosed and/or received their first course of treatment at the study hospitals of interest, which effectively excluded patients receiving irregular care following diagnosis at an outside institution. To explore whether the change in proportion of hormone receptor negative and advanced pathologic stage cancers was due to an increase in the number of those cancers or a decrease in the number of other types of breast cancers (e.g., hormone receptor positive), we plotted the case counts of each breast cancer type.

Analyses were performed using SAS software version 9.4 (SAS Institute Inc., Cary, NC) and statistical tests were two‐sided. *p* ≤ 0.05 were considered statistically significant.

## 3 RESULTS

### 3.1 Incidence trends

During the study time period, 9489 breast cancers, 958 colon cancers, 669 pancreatic cancers, and 1991 uterine cancers were diagnosed at the study hospitals (Table 1). The average age at diagnosis ranged from 60.2 (SD 13.0, breast cancer) to 68.5 (SD 11.8, pancreatic cancer). Across all cancer sites, the majority of patients were Black (23% to 27%) or White (69% to 72%); 2% to 4% of patients reported Hispanic ethnicity.

**TABLE 1:** Characteristics of breast, colon, pancreatic, and uterine cancer patients at three hospitals in a North Carolina health system, 2016–2020.

	Breast cancer	Colon cancer	Pancreatic cancer	Uterine cancer
	(*N* = 9489)	(*N* = 958)	(*N* = 669)	(*N* = 1991)
*Characteristic*	No. (%)^a^	No. (%)^a^	No. (%)^a^	No. (%)^a^
Age at diagnosis (years)				
<40	548 (6)	55 (6)	12 (2)	98 (5)
40–49	1567 (17)	100 (10)	23 (3)	152 (8)
50–59	2307 (24)	177 (18)	101 (15)	514 (26)
60–69	2695 (28)	228 (24)	206 (31)	669 (34)
70–79	1758 (19)	220 (23)	205 (31)	438 (22)
≥80	614 (6)	178 (19)	122 (18)	120 (6)
Race				
American Indian/Alaska Native	26 (<1)	<10 (<1)	<10 (<1)	<10 (<1)
Asian/Pacific Islander	222 (2)	14 (1)	13 (2)	36 (2)
Black	2163 (23)	239 (25)	182 (27)	474 (24)
Multiracial	21 (<1)	<10 (<1)	0	<10 (<1)
Other race	159 (2)	12 (1)	<10 (1)	35 (2)
White	6813 (72)	680 (71)	461 (69)	1405 (71)
Unknown	85	7	3	31
Ethnicity				
Hispanic or Latino	302 (3)	37 (4)	15 (2)	80 (4)
Not Hispanic or Latino	8803 (97)	904 (96)	642 (98)	1869 (96)
Unknown	384	17	12	42
Class of case				
Analytic	8819 (93)	819 (86)	563 (84)	1824 (92)
Non‐analytic	667 (7)	138 (14)	106 (16)	166 (8)
Unknown	3	1		1
AJCC clinical stage				
0	1489 (17)	27 (8)	<10 (<1)	<10 (1)
I	4518 (51)	42 (12)	149 (25)	515 (68)
II	1752 (20)	20 (6)	97 (16)	37 (5)
III	584 (7)	39 (11)	73 (12)	60 (8)
IV	445 (5)	221 (63)	282 (47)	134 (18)
Unknown	701	609	67	1241
AJCC pathologic stage^b^				
0	1265 (18)	15 (2)	0	<10 (<1)
I	4121 (58)	138 (18)	21 (10)	1054 (71)
II	1122 (16)	198 (25)	63 (29)	70 (5)
III	302 (4)	235 (30)	<10 (2)	234 (16)
IV	257 (4)	193 (25)	129 (59)	115 (8)
Unknown	1620	165	411	477
Tumor size (mm)				
<10	2140 (24)	47 (6)	<10 (1)	100 (7)
10 to <20	3015 (33)	57 (7)	52 (9)	152 (11)
20 to <30	1625 (18)	81 (10)	145 (24)	226 (16)
30 to <40	799 (9)	131 (17)	156 (26)	252 (18)
40 to <50	453 (5)	126 (16)	108 (18)	216 (15)
≥50	977 (11)	338 (43)	133 (22)	466 (33)
Unknown	480	178	69	579
Lymph node status				
Not evaluated	1909 (22)	203 (22)	507 (77)	603 (31)
Evaluated	6774 (78)	740 (78)	149 (23)	1363 (69)
Negative	4888 (72)	421 (57)	66 (44)	1148 (84)
Positive	1886 (28)	319 (43)	83 (56)	214 (16)
Unknown if evaluated	806	15		
*Breast cancer‐specific disease characteristics*				
ER		Not applicable		
Positive	7599 (82)			
Negative	1674 (18)			
Unknown	216			
PR				
Positive	6636 (72)			
Negative	2617 (28)			
Unknown	236			
HER2^c^				
Positive	1187 (15)			
Negative	6614 (85)			
Unknown	270			
Triple‐negative^c^				
Yes	1010 (13)			
No	6860 (87)			
Unknown	201			
AJCC pathologic prognostic stage				
0	1334 (20)			
I	4561 (68)			
II	431 (6)			
III	169 (3)			
IV	242 (4)			
Unknown	1449			

^a^ Calculated among observations with non‐missing values.

^b^ Among those not treated with neoadjuvant therapy.

^c^ Among invasive breast cancers only.

**FIGURE 1:**
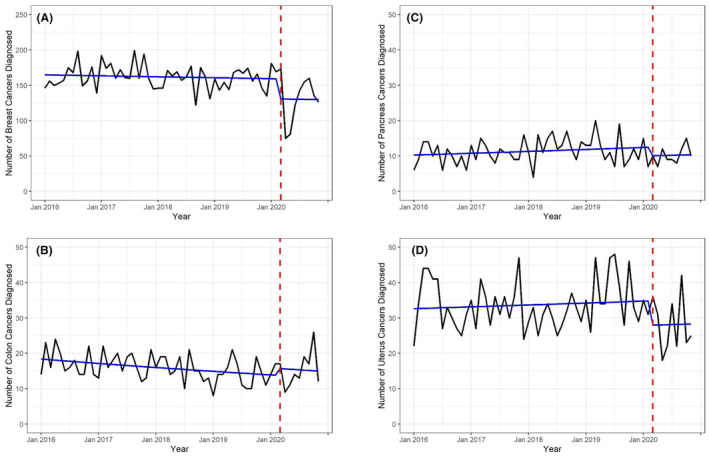
Number of incident cancers diagnosed between January 2016 and November 2020. The y‐axis reflects the total number of individuals diagnosed and the x‐axis reflects the time between January 2016 and November 2020. Raw case counts are shown in black and model‐based predicted case counts are shown in blue. The red dashed vertical line marks March 2020, representing the onset of the COVID‐19 pandemic in the US. (A) breast cancer, (B) colon cancer, (C) pancreatic cancer, (D) uterine cancer.

Prior to March 2020, the incidence of breast cancers and colon cancers was declining, whereas incidence of pancreatic and uterine cancers was increasing (Figure 1). Between March 2020 and November 2020, there was a statistically significant reduction in the number of new breast cancer diagnoses, with cancer incidence 18% lower than expected based on the pre‐pandemic trend between January 2016 and February 2020 (Table 2). Similarly, incidence of uterine cancer was 20% lower than expected between March 2020 and November 2020 (Table 2). The reduction in pancreatic cancers was of a similar magnitude but not statistically significant (Table 2). There was no reduction in the number of colon cancers diagnosed following the pandemic onset (Table 2). For all cancers, the results were similar to the main analysis when the pandemic period was restricted to March 2020 to August 2020, the date of the pandemic start was defined as April 1, 2020 instead of March 1, 2020, and when the analysis was restricted to cases diagnosed at and/or treated at the study hospitals (Figure S1; Table S2).

**TABLE 2:** Estimated monthly rate of change in cancer incidence in a North Carolina health system during the COVID‐19 pandemic.

Cancer site	Average monthly rate of change in cancer incidence (95% CI)^a^	*p*‐value^b^
Breast	−18% (−31%, −2%)	0.03
Colon	14% (−11%, 47%)	0.30
Pancreas	−20% (−37%, 3%)	0.08
Uterus	−20% (−36%, 0%)	0.05

^a^ Comparing the pandemic period (March 2020–November 2020) to the expected incidence based on the pre‐pandemic period (January 2016–February 2020).

^b^ *p*‐value, estimated with robust variance estimator.

### 3.2 Change in cancer types

**FIGURE 2:**
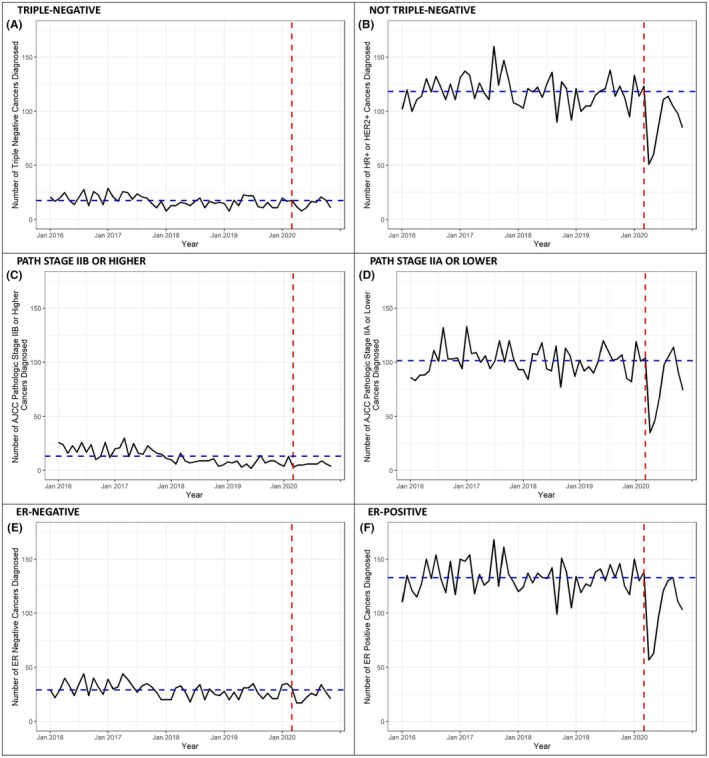
Incidence of poor prognosis breast cancers between January 2016 and November 2020. The y‐axis reflects the total number of individuals diagnosed and the x‐axis reflects the time between January 2016 and November 2020. Raw case counts are shown in black and model‐based predicted case counts are shown in blue. The red dashed vertical line marks March 2020, representing the onset of the COVID‐19 pandemic in the US. (A) triple‐negative, (B) hormone receptor and/or HER2‐positive, (C) pathologic stage IIB or higher, (D) pathologic stage IIA or lower, (E) estrogen receptor‐negative, (F) estrogen receptor‐positive.

To better understand factors driving the reduction in breast and uterine cancer incidence following the onset of the pandemic, we evaluated the prevalence of specific poor prognosis cancer types in pre‐pandemic versus pandemic periods. We found that the proportion of breast cancers that were pathologic stage IIB or higher increased significantly during the pandemic compared to what would have been expected based on pre‐pandemic trends (+4.02%, 95% CI 1.07–6.97; *p* = 0.01), as well as the proportion of breast cancers that were ER‐negative (+3.57%, 95% CI 0.87–6.28; *p* = 0.01) and triple‐negative (+3.55%, 95% CI 0.95–6.15; *p* = 0.01). As shown in Figure 2, there was no substantial increase in the number of ER‐negative, triple‐negative, or pathologic stage IIB or higher breast cancers and the change in proportion was driven by the decrease in receptor positive and lower stage tumors. No difference was detected in the proportion of other poor prognosis features including advanced (pathologic prognostic stage IIA or higher), clinical stage IIB or higher, >34 mm, lymph node‐positive, PR‐negative, or HER2‐positive breast cancers (all *p* > 0.05; Table 3). The finding that the proportions of lymph node positive and larger breast cancers were unchanged following the pandemic onset was consistent when tumors were stratified by ER status (Table S3).

**TABLE 3:** Estimated change in proportion of cancers with a poor prognostic characteristic in a North Carolina health system during the COVID‐19 pandemic.

Prognostic characteristic	Percent change, comparing March–November 2020 to January 2016–February 2020 (95% CI)	*p*‐value
Breast cancer		
Advanced breast cancer^a^ ^,^ ^b^	−0.1 (−2.8, 2.7)	0.96
Clinical stage IIB or higher	1.6 (−2.1, 5.2)	0.41
Pathologic stage IIB or higher^b^	4.0 (1.1, 7.0)	0.01
Estrogen receptor negative	3.6 (0.9, 6.3)	0.01
Progesterone receptor negative	1.4 (−1.7, 4.5)	0.38
HER2‐positive^c^	0.2 (−2.6, 3.0)	0.87
Triple‐negative^c^ ^,^ ^d^	3.6 (1.0, 6.2)	0.01
Tumor size >34 mm	0.5 (−2.3, 3.3)	0.74
Lymph node positive^e^	0.7 (−3.6, 4.9)	0.76
Uterine cancer		
Clinical stage II or higher	16.2 (5.4, 27.1)	<0.01
Pathologic stage II or higher^b^	6.5 (−2.3, 15.3)	0.15
Tumor size >62 mm	8.2 (0.7, 15.6)	0.03
Lymph node positive^e^	3.1 (−5.1, 11.2)	0.46

^a^ AJCC (8th edition) pathologic prognostic stage II or higher.

^b^ Among patients not treated with neoadjuvant therapy.

^c^ Among invasive tumors only.

^d^ ER‐negative, PR‐negative, and HER2‐negative.

^e^ Among patients for whom lymph nodes were evaluated.

The proportion of uterine cancers with an advanced clinical stage and large tumor size both showed statistically significant increases (clinical stage—+16.21%, 95% CI 5.36, 27.07; tumor size—+8.15%, 95% CI 0.67, 15.63), but there was no significant difference in the proportion of tumors with advanced pathologic stage or that were lymph node positive (Table 3). Similar to breast cancer, changes in proportions were driven by decreases in incidence of earlier stage and smaller uterine tumors (Figure S2). Graphical analyses showed no substantial increase in later clinical stage and larger uterine tumors during the pandemic when compared to incidence patterns prior to the pandemic.

## 4 DISCUSSION

Deferred screening exams can shorten a cancer's lead time (time between detection and onset of symptoms) and such delays may result in population‐level changes to cancer prognostic characteristics. Pre‐pandemic studies reported that increases in the breast cancer screening interval of just 1 year are associated with increased rates of tumors that are large (>15 mm), advanced stage at diagnosis, and high‐grade, all factors that are associated with increased morbidity and mortality.^20^, ^21^, ^22^, ^23^, ^24^ Similarly, delays in guideline‐concordant colon cancer screening are likely to have a wide range of impacts on late‐stage colon cancer incidence, ranging from 1% for a 3–6 month delay in surveillance colonoscopy to 20% for a 6 month delay in fecal immunohistochemical test (FIT) screening after a normal FIT test and an estimated increase of 0.3% in lifetime cancer incidence of for every month of delay in receiving colonoscopy after a positive FIT test.^25^, ^26^ Thus, pandemic‐related lead time reductions may contribute to a greater proportion of cancers diagnosed at a later stage, higher grade, and larger size. Further, diagnostic delays and resultant delays in therapy initiation allow additional time for symptomatic cancers to spread; these cancers are already more likely to be larger, higher grade, and hormone receptor negative than screen‐detected cancers. By some estimates, screening and diagnostic delays are expected to contribute to 2500–5000 excess breast cancer deaths and >4000 colorectal cancer deaths over 10 years.^27^, ^28^, ^29^ Though compelling, these estimates were made at the beginning of the pandemic, prior to the time when the magnitude of changes in cancer care and the impact on new diagnoses was known.

In our analysis of cancer diagnosis patterns in women at three large hospitals in a North Carolina health system, we found that the breast cancer incidence was reduced following the pandemic. The reduction in breast cancer diagnoses was similar to reports from other US regions^16^, ^30^, ^31^ and international sites.^32^, ^33^, ^34^, ^35^ The reduction in breast cancer incidence occurred between March 2020 and August 2020, mirroring steep drops in screening and diagnostic mammography that occurred during that time.^8^ We also found that the profile of breast cancers diagnosed during the pandemic differed from breast cancers diagnosed pre‐pandemic. The higher proportion of ER‐negative and triple‐negative tumors likely reflects the immediate effects of a reduction in screening‐detected ER‐positive tumors. Given that ER‐positive tumors are often slow‐growing, it is possible that the delayed diagnosis of these tumors may not lead to clinically significant differences in outcomes, assuming that a return to pre‐pandemic mammography patterns^36^ is able to detect cancers before they become advanced. Surveillance studies are needed to determine whether the long‐term prognosis for ER‐positive patients diagnosed during and shortly after the pandemic differs from historical ER‐positive breast cancer cohorts. The incidence curve also suggests that the decline in breast cancer cases reached a low point and was approaching pre‐pandemic levels toward the end of the study period. Studies of cancer trends into 2021 and beyond are needed to determine whether the cases that went undiagnosed in 2020 will be diagnosed at a later time.

We did not observe any changes in colon cancer incidence during the pandemic. Point estimates vacillated above and below zero when the pandemic time periods were defined differently; however the CIs always included 0, suggesting that estimates did not differ statistically from 0. This was in contrast to declines reported by other studies.^9^, ^14^, ^16^, ^30^, ^31^, ^32^, ^33^, ^37^ There are two main factors that may have contributed to differences between our study and others. First, it is possible that guideline‐concordant colon cancer screening may not have declined as much within our health system as in other systems or geographic areas. Although screening endoscopy was suspended at the beginning of the pandemic, there are multiple ways to screen for colon cancer such as using FIT, a test that can be performed without an office visit. In a nationally‐representative sample, a small decrease in screening colonoscopy use was balanced by an increase in the use of in‐home stool screening tests.^38^ Although positive stool tests require diagnostic confirmation by colonoscopy, patients with a positive stool test may have been more motivated to seek additional diagnostic care compared with patients requiring an office visit for an initial screening. Second, our study included only women, whereas other studies of colon cancer incidence during the pandemic included women and men. Women are more likely than men to seek out health information than men.^39^ It is possible that women were more likely to seek out screening and diagnostic care during the pandemic and therefore may not have experienced incidence reductions in the same way that men did. Additional studies that show results separately for men and women are needed to understand how gender may have impacted pandemic‐associated changes in cancer incidence. The observed reduction in uterine cancer is consistent with a previously reported 7.9% reduction.^16^ We also observed increases in the proportion of poorer prognosis uterine cancers driven by a reduction in earlier clinical stage and smaller tumors, which suggests that women with minimal or less severe symptoms may have been less likely to seek care during the pandemic. However, given that there was no difference in pathologic stage, it is unclear what effect the changes in clinical stage will have on long‐term outcomes for uterine cancer, if any. There were no detectable changes in pancreatic cancer, although statistical power may have been limited due to the overall low number of pancreatic cancer cases diagnosed each year. Though not statistically significant, the point estimate for the decline in pancreatic cancers in this study (20% decline) was similar to that reported in two national samples (24% decline).^14^, ^31^

In general, there was a dearth of published data on uterine^16^ and pancreatic^14^, ^30^, ^31^, ^34^ cancer trends, whereas almost all studies reported data on breast and colon or colorectal cancers.^9^, ^15^, ^30^, ^32^, ^33^, ^37^ This may be a natural consequence of prior reports describing large reductions in mammography and colonoscopy screening in 2020. However, it is essential that epidemiologic surveillance be conducted for all cancers. In particular, uterine cancer rates have been increasing steadily since 2006^40^ and, due to a lack of screening, is unlikely to be affected by potential overdiagnosis. As such, the interruption in uterine cancer cases is more likely reflective of delayed diagnoses that will result in larger and later stage tumors upon diagnosis, further compounding the problems posed by increasing incidence.

The impact of these trends on cancer incidence and the prognosis for those patients remains to be seen. Although our findings differ from other published reports, particularly with respect to colon cancer, the characteristics of pandemic‐related lockdowns, limitations on screening and other physician care availability varied across states and health systems. Accordingly, it has been noted that the screening recovery also varies by cancer site and geographic region.^41^ Furthermore, evaluations of screening patterns through 2022 suggest that there may be lingering periods of lower than expected screening rates among Medicare enrollees.^42^

These results should be interpreted in the context of several limitations. There were relatively low numbers of colon, uterine, and pancreatic cancers, which may have led to type I error. The study period included the first 9 months of the pandemic and does not reflect changes in cancer incidence that may have occurred after that time. Thus, we may not have been able to detect changes in the distribution of some tumor characteristics that take a longer time to become apparent. There were relatively high proportions of missing data for pathologic stage and prognostic pathologic stage, which could contribute to potential selection bias in analyses involving those characteristics. Additionally, this study was conducted using data from hospitals belonging to a single health system and may not be generalizable to other health care systems. These limitations are balanced by several strengths. The study hospitals are public, state‐funded entities with academic and community‐based providers and serve a socially and economically diverse population. It is likely that the results are generalizable to the population of North Carolina. Study data were obtained from hospital tumor registries and case characteristics abstracted by certified tumor registrar, which is the gold standard for cancer reporting. This enabled us to build on prior reports by addressing characteristics of the cancers for which incidence was reduced, which, to our knowledge, has not been previously reported in detail.

## 5 CONCLUSION

We found that the pandemic resulted in expected reductions in breast and uterine cancer incidence, but not the expected reductions in colon or pancreatic cancer incidence. Breast cancer reductions were largely due to a reduction in ER‐positive disease, which is consistent with a reduction in screen‐detected tumors. At the onset of the pandemic, health systems pivoted to serving highest acuity patients as required by public health limitations and workforce constraints. The trends reported here reflect the effects of that short‐term change as well as short and long‐term changes in patient willingness and ability to obtain cancer screening and diagnostic testing. These results reflect trends in the first 9 months of the pandemic, and likely vary in settings that had different cancer screening and/or COVID‐19 control procedures. Additional changes in incidence, tumor types, and mortality may become apparent in coming years.

## AUTHOR CONTRIBUTIONS

**Sarah J. Nyante:** Conceptualization (equal); data curation (supporting); formal analysis (equal); funding acquisition (lead); project administration (lead); supervision (lead); writing – original draft (lead); writing – review and editing (lead). **Allison Deal:** Conceptualization (equal); formal analysis (lead); funding acquisition (supporting); methodology (lead); supervision (supporting); validation (lead); writing – original draft (supporting); writing – review and editing (supporting). **Hillary M. Heiling:** Data curation (supporting); formal analysis (supporting); writing – original draft (supporting); writing – review and editing (supporting). **Kyung Su Kim:** Data curation (lead); writing – review and editing (supporting). **Cherie M. Kuzmiak:** Conceptualization (supporting); writing – original draft (supporting); writing – review and editing (supporting). **Benjamin C. Calhoun:** Conceptualization (supporting); writing – original draft (supporting); writing – review and editing (supporting). **Emily Ray:** Conceptualization (supporting); writing – original draft (supporting); writing – review and editing (supporting).

## FUNDING INFORMATION

National Cancer Institute grant R03CA267469.

## CONFLICT OF INTEREST STATEMENT

Dr. Calhoun has received consultation fees from PathAI. Dr. Ray reports research funding from OptumHealth and Pfizer, paid to her institution. The other authors have no potential conflicts of interest to disclose.

## ETHICS STATEMENT

This study was conducted in accordance with the principles of the Declaration of Helsinki. The study was approved by the University of North Carolina at Chapel Hill Institutional Review Board (IRB# 21‐1708) with a waiver of informed consent and waiver of HIPAA approval.

## Supporting information


Figure S1.

Figure S2.

Table S1.

Table S2.

Table S3.

## Data Availability

The data underlying study article were provided by hospitals in the University of North Carolina Health system under a data use agreement. Data will be shared by the corresponding author pending permission from the University of North Carolina Health system.
