# Supplementary material for: Trends in breast, colon, pancreatic, and uterine cancers in women during the COVID‐19 pandemic in North Carolina
Source: Cancer Med. 2024 Apr 4;13(7):e7156. doi: 10.1002/cam4.7156 (PMC10993709; doi:10.1002/cam4.7156)
Supplement: Supplementary file 1 — Figure S1. [file CAM4-13-e7156-s001.pdf]

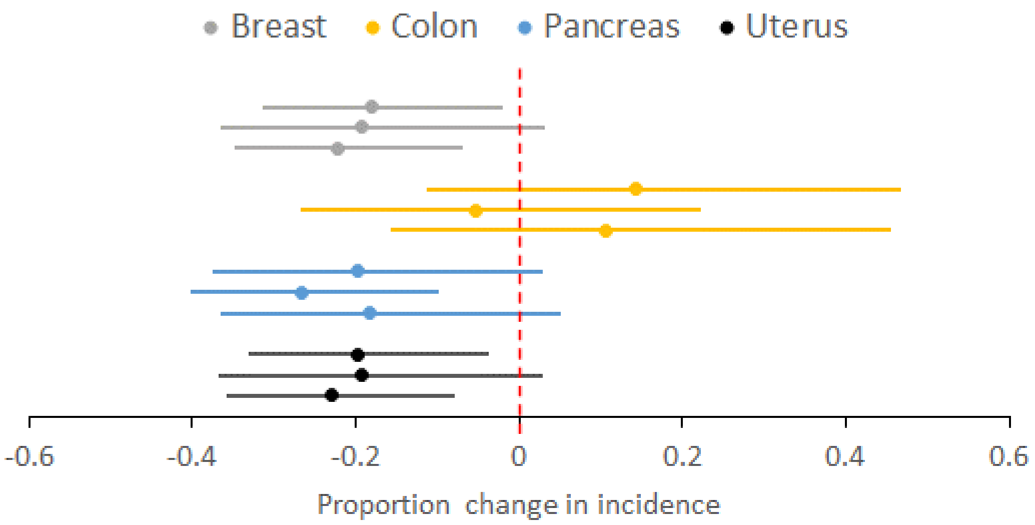

Supplementary Figure S1. Proportional monthly change in cancer incidence under varying definitions of the pandemic time period. For each cancer site, the proportional monthly change in incidence during the pandemic compared with before the pandemic is shown. For each cancer site, the three lines represent different definitions of the pandemic period. Estimates in the top line correspond to the pandemic defined as March 2020 – November 2020 (used in the main analysis); the middle line corresponds to the pandemic defined as March 2020 – August 2020; and the bottom line corresponds to the pandemic defined as April 2020 – November 2020. Error bars represent the 95% confidence interval.
