# Supplementary material for: Trends in breast, colon, pancreatic, and uterine cancers in women during the COVID‐19 pandemic in North Carolina
Source: Cancer Med. 2024 Apr 4;13(7):e7156. doi: 10.1002/cam4.7156 (PMC10993709; doi:10.1002/cam4.7156)
Supplement: Supplementary file 2 — Figure S2. [file CAM4-13-e7156-s005.pdf]

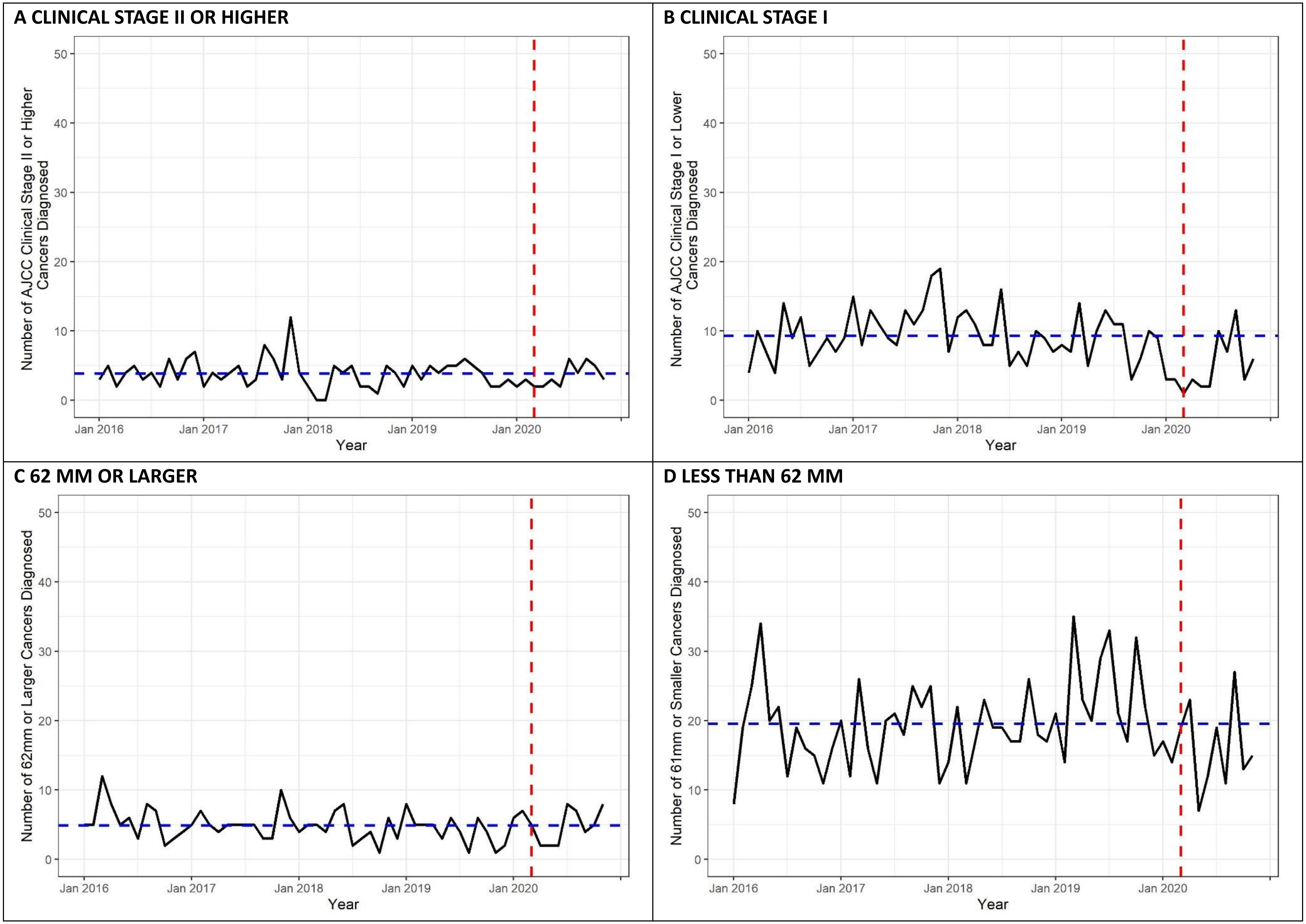
A CLINICAL STAGE II OR HIGHER
Number of AJCC Clinical Stage II or Higher Cancers Diagnosed
Year
Jan 2016
Jan 2017
Jan 2018
Jan 2019
Jan 2020
B CLINICAL STAGE I
Number of AJCC Clinical Stage I or Lower Cancers Diagnosed
C 62 MM OR LARGER
Number of 62mm or Larger Cancers Diagnosed
D LESS THAN 62 MM
Number of 61mm or Smaller Cancers Diagnosed

Supplementary Figure S2. Incidence of poor prognosis uterine cancers between January 2016 and November 2020. The y-axis reflects the total number of individuals diagnosed and the x-axis reflects the time between January 2016 and November 2020. Raw case counts are shown in black and model-based predicted case counts are shown in blue. The red dashed vertical line marks March 2020, representing the onset of the Covid-19 pandemic in the US. Panel A – clinical stage II or higher, panel B – clinical stage I, panel C – 62 mm or larger in size, panel D – less than 62 mm in size.
