# Supplementary material for: Trends in breast, colon, pancreatic, and uterine cancers in women during the COVID‐19 pandemic in North Carolina
Source: Cancer Med. 2024 Apr 4;13(7):e7156. doi: 10.1002/cam4.7156 (PMC10993709; doi:10.1002/cam4.7156)
Supplement: Supplementary file 3 — Table S1. [file CAM4-13-e7156-s004.docx]

**Supplementary Table S1.** Selected North Carolina executive actions related to Covid-19 in 2020.

| Date Issued | State Guidance |
| --- | --- |
| March 10 | State of emergency declared (executive order 116) |
| March 12 | Cancel or postpone gatherings of >100 people; telework if possible |
| March 14 | K-12 schools to close; ban on gatherings of >100 people in a single space (executive order 117) |
| March 23 | Entertainment facilities without retail or dining, personal care and grooming businesses to close (executive order 120) |
| March 27 | State-wide Stay At Home order issued (executive order 121) |
| May 6 | NC moves to Phase 1 of easing Covid restrictions; certain non-essential retail businesses allowed to open at 50% capacity; small outdoor gatherings allowed (executive order 138) |
| May 20 | NC moves to Phase 2 of Covid response: Safer-At-Home (executive order 141) |
| July 14 | Health and safety plan released for in-person K-12 instruction |
| September 4 | NC moves to Phase 2.5 of Covid response, increasing mass gathering limits and allowing some recreational facilities to open at reduced capacity (executive order 163) |
| September 30 | NC moves to Phase 3 of Covid response, allowing outdoor venues, movie theaters, bars, amusement parks to operate at reduced capacity (executive order 169) |
