# Supplementary material for: Trends in breast, colon, pancreatic, and uterine cancers in women during the COVID‐19 pandemic in North Carolina
Source: Cancer Med. 2024 Apr 4;13(7):e7156. doi: 10.1002/cam4.7156 (PMC10993709; doi:10.1002/cam4.7156)
Supplement: Supplementary file 4 — Table S2. [file CAM4-13-e7156-s003.docx]

**Supplementary Table S2.** Estimated change in incidence of breast, colon, pancreatic, and uterine cancers among analytic^a^ cases only in a North Carolina health system during the Covid-19 pandemic compared to before the Covid-19 pandemic.

| **Cancer site** | **Average monthly rate of change^b^ in cancer incidence (95% CI)** | **P-value^c^** |
| --- | --- | --- |
| Breast | -18% (-31%, -1%) | 0.04 |
| Colon | 16% (-12%, 52%) | 0.29 |
| Pancreas | -5% (-25%, 19%) | 0.65 |
| Uterus | -17% (-33%, 4%) | 0.11 |

^a^Individuals who were diagnosed and/or received their first course of treatment at the study hospitals of interest

^b^Comparing the pandemic period (March 2020-November 2020) with to expected incidence based on the pre-pandemic period (January 2016-February 2020)

^c^P-value, estimated with robust variance estimator
