# Supplementary material for: Trends in breast, colon, pancreatic, and uterine cancers in women during the COVID‐19 pandemic in North Carolina
Source: Cancer Med. 2024 Apr 4;13(7):e7156. doi: 10.1002/cam4.7156 (PMC10993709; doi:10.1002/cam4.7156)
Supplement: Supplementary file 5 — Table S3. [file CAM4-13-e7156-s002.docx]

**Supplementary Table S3.** Estimates of the change in proportion of breast cancers with a poor prognostic characteristic according to estrogen receptor status, comparing cancers diagnosed between March 2020 and November 2020 with cancers diagnosed between January 2016 and February.

| **Breast cancer type** | **Prognostic characteristic** | **Percent change comparing March-November 2020 to January 2016-February 2020 (95% CI)** | **P-value** |
| --- | --- | --- | --- |
| ER^a^-positive | Lymph node positive^b^ | 0.8 (-4.0, 5.7) | 0.73 |
|  | Tumour size > 34 mm | -0.3 (-3.1, 2.5) | 0.82 |
|  |  |  |  |
| ER^a^-negative | Lymph node positive^b^ | -1.0 (-10.4, 8.5) | 0.84 |
|  | Tumour size > 34 mm | 1.7 (-6.0, 9.4) | 0.67 |

^a^ER – estrogen receptor

^b^Among patients for whom lymph nodes were evaluated
